# Seroprevalence of leptospiral infection in feline population in urban and dairy cattle herds in Mashhad, Iran

**Published:** 2015-12-15

**Authors:** Massoud Talebkhan Garoussi, Mohsen Mehravaran, Gholamreza Abdollahpour, Javad Khoshnegah

**Affiliations:** 1 *Department of Theriogenology, Faculty of Veterinary Medicine, University of Tehran, Tehran, Iran; *; 2 *Department of Clinical Sciences, Faculty of Veterinary Medicine, Ferdowsi University of Mashhad, Mashhad, Iran; *; 3 *Leptospira Laboratory Research Center and Department of Internal Medicine, Faculty of Veterinary Medicine, University of Tehran, Iran.*

**Keywords:** Cat, Iran, *Leptospira*, Mashhad, Sero-epidemiology

## Abstract

The importance of cats in the *Leptospira* epidemiology is due to the possibility of transferring leptospirosis to wild and domesticated animals. The purpose of this survey was to determine the prevalence of *Leptospira *infection in shorthair cats in different location of Mashhad, Iran. Totally, 147 blood samples were taken from 42 (28.57%), 52 (35.37%) and 53 (36.05%) households, stray and cats which lived in industrial dairy cattle herds of Mashhad, Iran, respectively. Sera were tested with seven live *Leptospira* antigens using microscopic agglutination test (MAT). Sera with 50.00% agglutination at the dilution of ≥ 1/100 were considered as positive samples. Agglutination at dilutions of < 1/100 considered as suspected to *Leptospira* infection. Overall, 19 (12.92%) out of 147 cats showed reaction in MAT. The seroprevalence at a titer ≥ 1:100 and < 1:100 were 10 (6.80%) and 9 (6.12%), respectively. Serum samples showed positive reaction against *Leptospira* *intterogans hardjo* (no = 10; 52.63%), *pomona* (no = 5; 26.31%) and *icterohaemorrhagiae *(no = 4; 21.05%). Eight cats (42.10%) belong to dairy cattle herds had the most infection only by *L. I. hardjo* with 1:200 titer. There were no significant differences among the weight‚ age and sex of infected cats. However, there were significant differences between the infected cats in dairy cattle herds and the cats in the urban area (*p* < 0.05). It is concluded that cats can be infected by *Leptospira* spp. especially in commercial dairy cattle herds. Cats can be considered as a sanitation hazards in the area for this zoonotic disease.

## Introduction

Leptospirosis is a zoonotic disease caused by various species of *Leptospira*, a spirochete in the family Leptospiraceae, order Spirochaetales. The pathogenic leptospires are classified into one species of *Leptospira interrogans* (*L. I*.) consist of 23 serogroups and 212 serovars.^1^ Leptospirosis has been reported world-wide and is prevalent in wild and domestic animals. The prevalence of clinical leptospirosis in cats is very low. They may be exposed to leptospires excreted by wildlife and domestic animals such as rodents and cows. Also cats may be exposed to infected urine of cohabitating dogs and rodents as the main carrier of leptospires. Sero-surveys generally show exposure rate about 10.00%. Outdoor cats have the highest seroprevalence of leptospires. Serovars *L. I. canicola, grippotyphosa* and *Pomona* have been isolated from cats.^2^ Clinical signs are usually mild or unapparent in feline leptospirosis despite of the presence of leptospiremia, leptospiruria and histologic evidence of renal and hepatic inflammation.^2^ Since cats are resistant to both natural and experimental leptospirosis, therefore, the clinical manifestations of leptospirosis are still controversial.^3^^,^^4^ Serological surveys and bacteriological evidences of leptospirosis in cats have been shown from different countries.^5^^-^^7^

Leptospiral infection in rodents, dairy cattle herds and shepherd dogs has been investigated in Mashhad, Iran.^8^^-^^10^ However, there are very few investigations in the field of leptospirosis in cats.

The aims of this survey was to determine the prevalence of *Leptospira* infection in shorthair cats population included households, stray and cats which lived in industrial dairy cattle herds in Mashhad, Iran.

## Material and Methods

**Geographical** **region** **and** **study** **population. **Mashhad, in Khorasan Razavi province, is a major producer of livestock in North-East and the second center of dairy production in Iran. Samples were taken randomly using a lottery mechanism. None of the herds and cats was vaccinated against leptospires.

The sample size required estimating the sero-prevalence of *Leptospira* in the population of shorthair cats in urban and in the dairy cattle herds, with level of confidence of 95.00%, desired absolute precision 5.00% and an expected prevalence of 10%, the minimum required sample size was 138 cats using the following formula:^11^

n =1.96 Pexp (1-Pexp)d2

where, *n *is required sample size; *P*_exp_ is expected pre-valence; *d* is desired absolute precision; 1.962 is multiplier.

The samples were collected from March 2008 to November 2010. Totally, 147 shorthair cats’ blood samples were obtained. The study population consisted of 42 (28.57%), 52 (35.37%) and 53 (36.05%), households, stray and also cats which captured from industrial dairy cattle herds in suburb of Mashhad, Iran, respectively. The owners of the herds and the cats were not informed for the aims of this study. Both stray cats and cats in industrial dairy cattle herds were captured using designed wooden cages. Cages were placed at different points that cats have passed. The captured cats transferred to the small animal hospital of faculty of veterinary medicine, Ferdowsi university of Mashhad, Iran. The cats were taken to the hospital for routine clinical examinations and blood sampling. Sixty-three (42.85%) of the animals were male and 84 (57.15%) were female. Age, weight and season of capture were recorded. ^12^

**Sample collection. **Intramuscular ketamine hydro-chloride (2.5 mg kg^-1^; Inverin Co., Galway, Ireland) and Acepromazine (0.25 mg kg^-1^; Kela Laboratoria, Hoogstraten, Belgium) were used for tranquillizing. Blood samples (5 mL) were obtained using venipuncture from cephalic vein. The samples were carried in ice bag to *Leptospira* Laboratory Research Center, Faculty of Veterinary Medicine, University of Tehran (Tehran, Iran). The collected sera were stored at – 20 ˚C untill analyzed.

**Assays. **Samples were assayed using microscopic agglutination test (MAT).^9^^,^^10^^,^^13^ Samples that had 50.00% agglutination reaction were considered as positive and sera with agglutination under 50.00% were considered as suspected. Samples were tested with seven live *Leptospira* antigens consist of *L.I. hardjo, L. I. icterohaemorrhagiae, L. I. pomona, L. I. grippotyphosa, L. I. autumnalis, L. I. canicola and L. I. ballum*. 

**Statistical analysis. **Data were analyzed with Pearson Chi-Square, using SPSS (version 16; SPSS Inc., Chicago, USA). For all analyses, a *p-*value less than 0.05 was considered as significant.

## Results

The result demonstrated that 19 (12.92%) out of 147 cats had positive reaction in MAT (positive serological reaction against *Leptospira* antigens). The age of sampled cats ranged from < 1 and > 2 years old (Table 1). However, there was no female cat more than 2 years old in dairy cattle herds. The maximum MAT titer was 1:200 for 9 (6.12%) and 1 (0.68%) infected cats in dairy cattle herds and stray cat, respectively (Table 1). However, 9 (6.12%) cats had < 1:100 titers in MAT that they were consider as suspected cases for leptospirosis (Table 1). The most common (no = 10; 52.63%) serovar was *L. I.* *hardjo *(Table 2). The cats in dairy cattle herds had the most infection (no = 8; 42.10%) with* L. I.* *hardjo* and 1:200 titer. The cats were also infected with *L. I.* *pomona* (no = 5; 26.31%) and *L. I. icterohaemorrhagiae *(no = 4; 21.05%).

**Table 1.** Sero-survey of *Leptospira* in shorthair cats using microscopic agglutination test (MAT) in Mashhad, Iran

**MAT**	**Number of cats (%)**				**Age (%)**			**Sex (%)**	
	**Household**	**Stray**	**Dairy cattle herd**	**Total**	**≤ 1 year**	**1 - 2 year**	**> 2 year**	**Male**	**Female**
**Positive**	-	1 (0.68)	9 (6.12)*	10 (6.8)	1 (0.68)	8 (5.44)	1 (0.68)	7 (4.76)	3 (2.04)
**Suspicious**	3 (2.04)	3 (2.04)	3 (2.04)	9 (6.12)	3 (2.04)	4 (2.72)	2 (1.36)	5 (3.40)	4 (2.72)
**Negative**	39 (26.53)	48 (32.65)	41 (27.89)	128 (87.07)	55 (37.41)	49 (33.33)	24 (16.32)	51 (34.69)	77 (52.38)
**Total**	42 (28.57)	52 (35.37)	53 (36.05)	147 (100.00)	59 (40.13)	61 (41.49)	27 (18.36)	63 (42.85)	84 (57.15)

* indicates significant difference among the groups (*p* < 0.05).

However, the cats in dairy cattle herds had the most infection and significant differences (Table 1). It was shown that *L. I. hardjo *was the most infected serovar (Table 2). All infected cats aged ≤ 1 (no = 4; 2.72%), 1 to 2 (no = 12; 8.16%) and > 2 (no = 3; 2.04%) years old (Table 1). The weight of five cats (3.40%) ranged 3 to 4 kg and two cats (1.36%) weighted 1 to 2 kg (Table 2).

**Table 2:** Distribution frequency of *Leptospira* serovars in shorthair cats located in different places in Mashhad, Iran.

**Serovars**	**Number of cats (%)**				**Weight (%)**				
	**Household**	**Stray**	**Dairy cattle herd**	**Total**	**≤ 1 kg **	**1 - 2 kg **	**2 - 3 kg **	**3 - 4 kg **	**> 4 kg **
***L. I. H***	1 (5.26)	1 (5.26)	8 (42.10)*	10 (52.63)	-	2 (1.36)	3 (0.68)	5 (3.40)	-
***L. I. P***	1 (5.26)	2 (10.52)	2 (10.52)	5 (26.31)	-	1 (0.68)	2 (1.36)	2(1.36)	-
***L. I. I***	1 (5.26)	1(5.26)	2 (10.52)	4 (21.05)	-	1 (0.68)	2 (1.36)	-	1 (0.68)
**Negative**	-	-	-	-	4 (2.72)	29 (19.72)	44 (29.93)	28 (19.04)	33 (22.44)
**Total**	3 (15.78)	4 (21.05)	12 (63.15)	19 (100.00)	4 (2.72)	31 (21.08)	45 (30.61)	33 (22.44)	34 (23.12)

*L. I. H*: *Leptospira interrogans hardjo*; *L. I. P*: *Leptospira interrogans pomona*; *L. I. I*: *Leptospira interrogans icterohaemorrhagiae*.

* indicates significant difference among the groups (*p* < 0.05).

## Discussion

There are a few investigations about feline *Leptospira* infection. This study showed that 12.92% of shorthair cats were infected with different *Leptospira* serovars in < 1/100 or = 1/200 dilutions. In other studies showed that more dilution such as 1/30 and 1/50 were considered positive.^14^ Since there is no routine vaccination program for cats’ population against leptospirosis in Mashhad, Khorasan Razavi province, Iran, these positive sera can be considered as active or previous infection. Isolation and serological surveys from various countries indicated that cats might be naturally exposed and subsequently infected by several *L. I.* serovars.^5^^,^^7^^,^^15^

The MAT is the most common serological test used for diagnosis of leptospirosis.^1^ Leptospiral infection may occur in the absence of detectable agglutination titer, unrelated and unknown or excluded serovars may be missed.^16^

It was shown a prevalence of 52.83% only with *Leptospira* serovar *hardjo *(Table 2). Eight out of 10 infected cats (42.10%) were from dairy cattle herds and infection could be acquired from cattle. Cattle are the main host of this serovar.^1^ There is no feline leptospirosis sero-epidemiological study in cats in dairy cattle herds in Iran or elsewhere. Therefore, this study seems to be important to determine the epidemiology of leptospirosis and the role of cat as a carrier in dairy farms. 

Animals can be divided into maintenance hosts or accidental (incidental) hosts.^17^ Since, cats are the natural hunter of different rodent species; thus, they may be considered as a reservoir of *Leptospira* infection. It was shown that rodents were infected by *L. I.* serogroups/ serovars *icterohaemorrhagiae, grippotyphosa* and *sejroe hardjo* in dairy cattle herds of Mashhad-Iran.^10^ The extent to which infection is transmitted depends upon many factors included: population densities, degree of contact between maintenance and accidental hosts. Since, population of mice in Mashhad city is not that huge, therefore, it may be the reason of lower seropositive cats versus others.^18^

Rats and dogs are the most important carrier of *Leptospira* in dairy herds and may have close exposure to cats.^8^^,^^10^ Cows can be an accidental host for *L. I. pomona* serovar. Only 1 (5.26%) household, 1 (5.26%) of stray cats and 2 (10.52%) cats in dairy cattle herds reacted against *L. I. pomona *(Table 2). This serovar can cause abortion and fatal hemolytic disease in calves.^1^

One (5.26%) of the household cats, one (5.26%) stray cat and 2 (10.52%) cats in dairy cattle herds reacted against *L. I. icterohaemorrhagiae* antigen (Table 2). Rats are the maintenance host of *icterohaemorrhagiae* and close exposure to rodents, wild life and stray dogs may be considered as the origin of the infection. Serological surveys from various countries showed that cats may be naturally exposed and subsequent infected by several *L. I.* serovars.^5^^,^^6^^,^^19^ Mice (*Mus musculus* and other *Mus* spp.) and rats (mainly *Rattus norvegicus*, *R. rattumole *and* Nesokia indica*) serve as reservoirs for *L. I. icterohaemorrhagiae, grippotyphosa, sejroe, *and* copenhagani.*^10^^,^^20^ Cats can acquire leptospires via these hosts. They usually do not show signs, however, harbour leptospires in their kidney, becoming an important source of infection for human or other domestic and wild animals. 

Cattle are the maintenance host of *L. I. hardjo*. Eight out of 10 *L. I.* *hardjo* infected cats were from dairy cattle herds (Table 2). It was shown that dairy cattle herds and the rodents in suburb of Mashhad, Iran were infected by these *Leptospira* serovars.^9^^,^^10^ However, infection in these cats could have been from cattle as the actual source of infection.

The role of age, sex, life style and contact with possible reservoir hosts as potential risk factors has not been adequately investigated. There was no association between seropositivity and the animals sex, age and body weight (Tables 1 and 2). However, most of the infected cats (no = 7; 36.84%) in industrial dairy cattle herds were male. It was found significant differences between body sizes and *Leptospira* seropositivity in rodents.^10^ It was found an association between age and seropositivity, as well.^5^ It may be due to physical body differences between male and female cats in dairy cattle herds. The body size of male cats may be bigger than females, this could increase the the chance of exposure to the contaminated food in male cats.

Knowledge of the prevalent serovars and the maintenance or accidental hosts are essential in understanding the epidemiology and implementing control of the disease in any region.

It is concluded that shorthair cat in different location, especially, cats in dairy cattle herds can be infected with different *Leptospira* serovars and shed them in the environment. Since, leptospirosis is a zoonotic disease, cats can be hazardous for public health in dairy cattle herds and the employees.
